# Supplementary material for: Colonization Ability of Bacillus subtilis NCD-2 in Different Crops and Its Effect on Rhizosphere Microorganisms
Source: Microorganisms. 2023 Mar 17;11(3):776. doi: 10.3390/microorganisms11030776 (PMC10058285; doi:10.3390/microorganisms11030776)

Figure S1 Ace curves of bacterial under different treatments


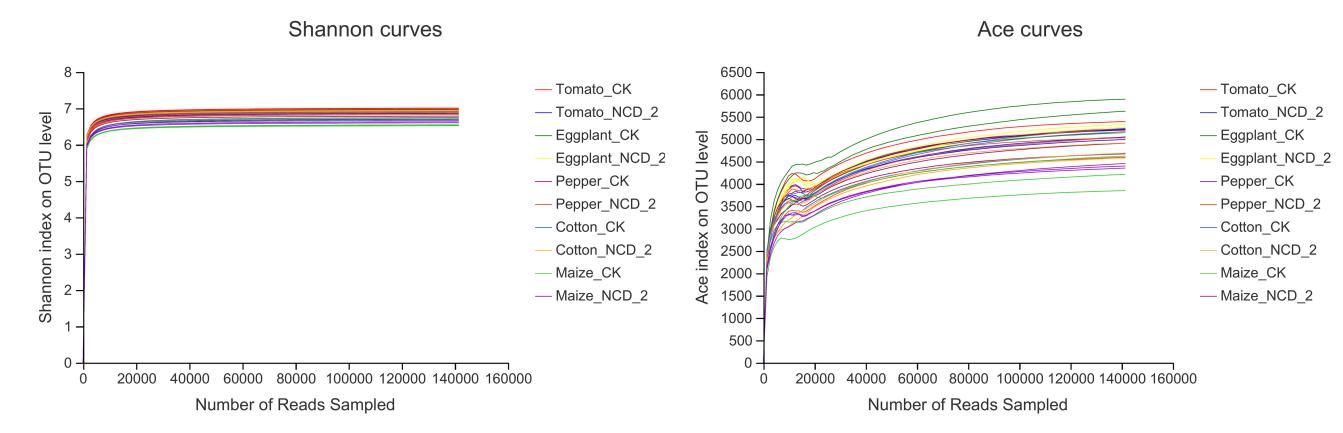


Figure S2 Ace curves of fungi under different treatments


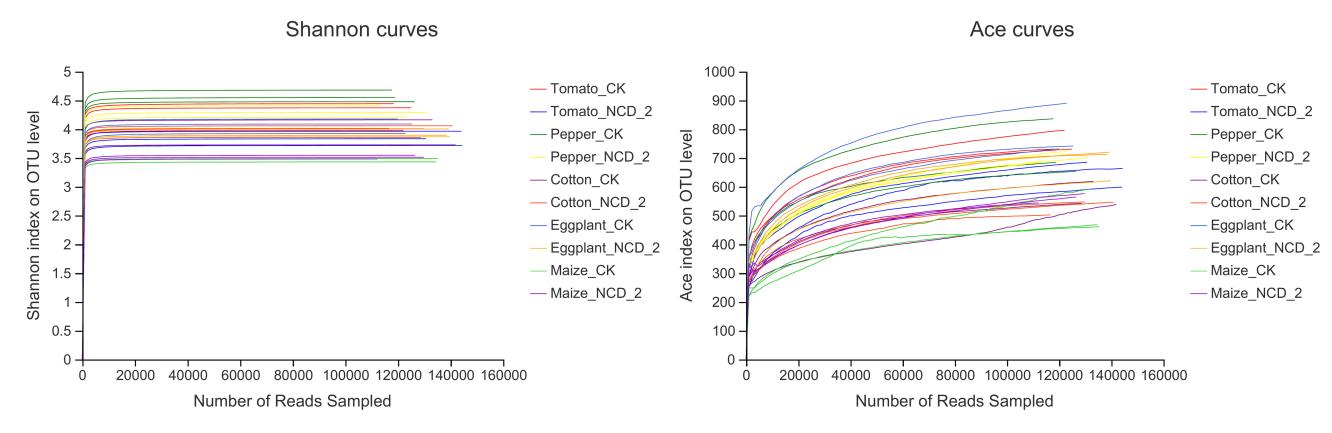


Figure S3 Venn diagram of unique and shared bacterial phyla in different treatments


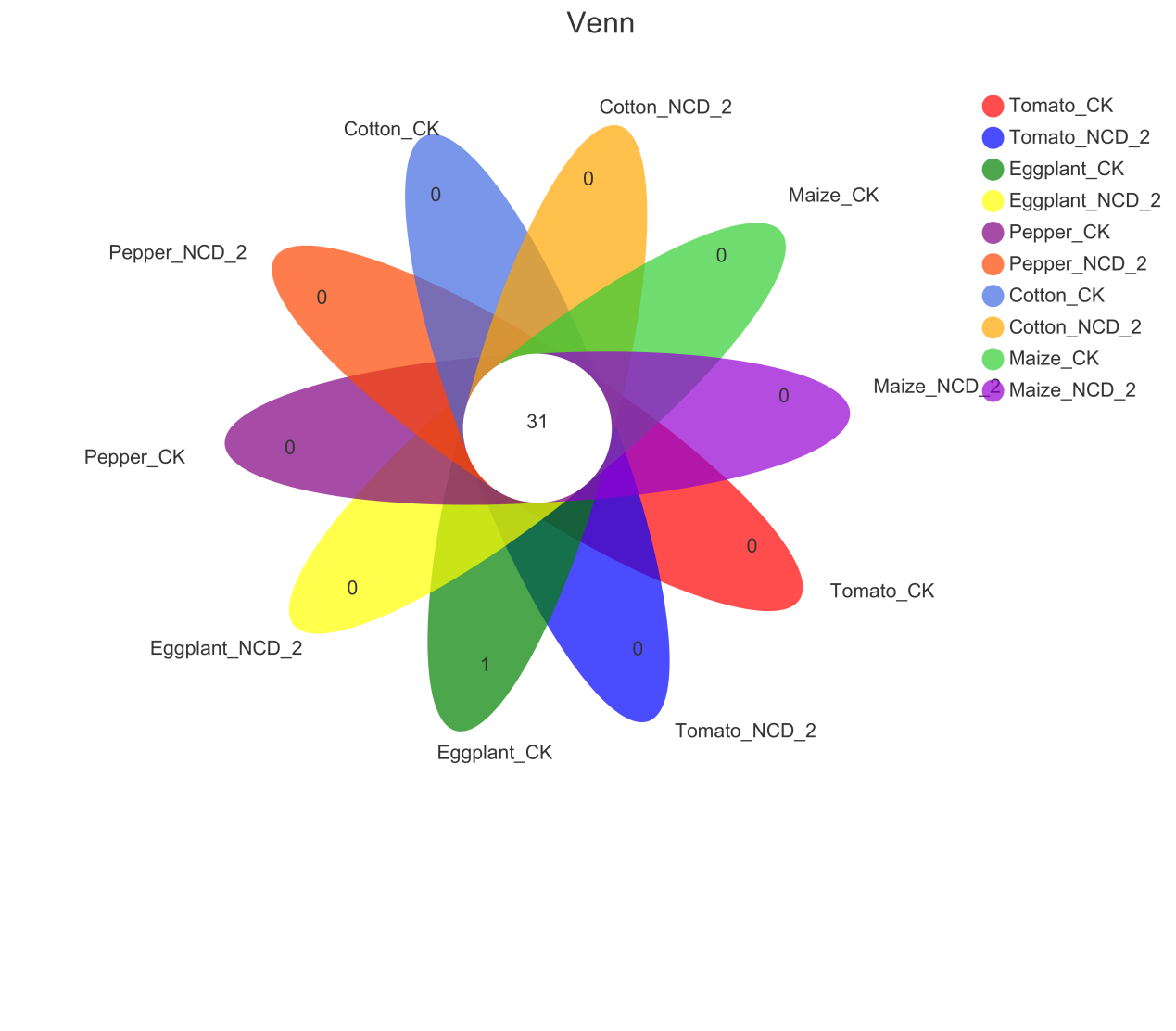

Supplement: Supplementary file 1 [file microorganisms-11-00776-s001.zip › Supplementary figure captions.docx]
